# A Highly Selective Biosensor Based on Peptide Directly Derived from the HarmOBP7 Aldehyde Binding Site

**DOI:** 10.3390/s19194284

**Published:** 2019-10-03

**Authors:** Tomasz Wasilewski, Bartosz Szulczyński, Marek Wojciechowski, Wojciech Kamysz, Jacek Gębicki

**Affiliations:** 1 Department of Inorganic Chemistry, Faculty of Pharmacy, Medical University of Gdańsk, Hallera 107, 80-416 Gdansk, Poland; kamysz@gumed.edu.pl; 2 Department of Process Engineering and Chemical Technology, Chemical Faculty, Gdańsk University of Technology, Narutowicza 11/12, 80-233 Gdańsk, Poland; bartosz.szulczynski@pg.edu.pl; 3 Department of Pharmaceutical Technology and Biochemistry, Chemical Faculty, Gdansk University of Technology, Narutowicza 11/12, 80-233 Gdańsk, Poland; marek.wojciechowski@pg.edu.pl

**Keywords:** sensors, biosensors, odorant binding proteins, olfactory receptors, bioelectronic nose, 33 peptides, quartz crystal microbalance

## Abstract

This paper presents the results of research on determining the optimal length of a peptide chain to effectively bind octanal molecules. Peptides that map the aldehyde binding site in HarmOBP7 were immobilized on piezoelectric transducers. Based on computational studies, four Odorant Binding Protein-derived Peptides (OBPPs) with different sequences were selected. Molecular modelling results of ligand docking with selected peptides were correlated with experimental results. The use of low-molecular synthetic peptides, instead of the whole protein, enabled the construction OBPPs-based biosensors. This work aims at developing a biomimetic piezoelectric OBPPs sensor for selective detection of octanal. Moreover, the research is concerned with the ligand binding affinity depending on different peptides’ chain lengths. The authors believe that the chain length can have a substantial influence on the type and effectiveness of peptide–ligand interaction. A confirmation of in silico investigation results is the correlation with the experimental results, which shows that the highest affinity to octanal is exhibited by the longest peptide (OBPP4 – KLLFDSLTDLKKKMSEC-NH_2_). We hypothesized that the binding of long chain aldehydes to the peptide, mimicking the binding site of HarmOBP7, induced a conformational change in the peptide deposited on a selected transducer. The constructed OBPP4-based biosensors were able to selectively bind octanal in the gas phase. It was also shown that the sensors were characterized by high selectivity with respect to octanal, as well as to acetaldehyde and benzaldehyde. The results indicate that the OBPP4 peptide, mimicking the binding domain in the Odorant Binding Protein, can provide new opportunities for the development of biomimicking materials in the field of odor biosensors.

## 1. Introduction

Recent years have witnessed a progress in biomaterials used as a receptor layer in odor sensors [1]. Development of biosensor technology is focused on achieving the parameters close to those of their biological counterparts [2]. A concept, which can fulfil these expectations, relies on utilization of the biological components from olfactory systems [3]. A better understanding of ligand affinity to the bioreceptor layer can result in a progress of odor biosensors. Key factors in production of the biosensors are a type of biosensing element and a technique of coupling with a transducer part, which have a direct impact on efficiency and operation parameters of the biosensor [4], capable to sensitively and selectively detect target compounds. Aldehydes contribute to the flavor of edible oils. Octanal represents the group of secondary oxidation products of oleic acid [5] and markers of food spoilage [6], and also was identified as biomarker of lung cancer when presented in breath condensates [7]. Apart from typical biological components of olfactory systems [8], odor biosensors employ structures mimicking biological materials, such as synthetic polypeptides [9]. The structure and activity of these materials are designed to mimic the odor molecules’ binding sites present in olfactory receptors. The investigations aimed at determination of the affinity of ligands to olfactory receptors (ORs) and to odorant binding proteins (OBPs) have been intensified. OBPs belong to the key elements of chemosensory systems [10] and possess substantial potential as biosensor elements [11,12,13]. Detection of natural and synthetic volatile organic compounds (VOCs) using OBPs has a high development potential [14]. However, their application in bioelectronic systems has just started to be developed [15]. Difficulties with implementation of biomaterials to biosensors construction are connected with their production and stability [3]. Therefore, methodological and equipment approaches, based on the application of artificial olfactory receptor sequences to the construction of biosensors, are being elaborated. Utilization of the protein components, such as ligand binding regions or synthetic peptides, belongs to new trends in analysis of simple odorous compounds with the biosensors [16]. The characteristic feature of the aforementioned applications is the fact that building sensors requires knowledge of binding pairs of olfactory receptors and suitable ligands, rather than the mechanisms of olfactory signal transmission [17]. Superior parameters of the peptide-based biosensor are expected for longer peptides because they can adopt multidimensional, stable structures upon interaction with ligands [18]. Moreover, attaining the random structure can be minimized via implementation of a rigid base bone structure, e.g., alpha-helix bundle [19] or cyclic ones [20]. The influence of the polypeptide chain length on fundamental parameters of the biosensors has not been unequivocally optimized as of yet. Typically, the peptides containing five to fifteen amino acid residues are used. This does not mean that more complex structures cannot be employed in the biosensors. However, the effect of sequence length must be determined to obtain high sensitivity and selectivity [21]. Polypeptides, as synthetic biological materials, possess many advantages owing to the fact that in a solution they reveal stable secondary structures, stabilized with hydrogen bonds. The results from ion mobility studies of tryptic peptides suggest that, in some cases, peptides in the gas phase structures can be related to the solution-phase structures [22]. In an alpha-helical synthetic polypeptide, parallel and directional alignment of hydrogen bonds along the helical axis collectively produces strong electric dipole moments and makes the peptide susceptible to electric and magnetic fields, which makes them suitable for biosensors. Further, site-specific functionalization can readily be obtained [23]. Moreover, if odorant/receptor binding regions can be identified, it is also possible to determine the peptide sequence, improving sensor parameters as far as specificity, repeatability, and sensitivity are concerned. Additionally, this contributes to a predictable output and lower production cost [24]. To effectively bind ligands, the receptor has to be characterized by appropriate spatial structure. Peptides are generally more flexible and are rarely characterized by secondary structures, usually occurring in the form of random coils. Only by interaction with the receptor/cell membrane or randomly for a short time, they can form an organized structure (e.g., alpha helix). Compounds planned for the synthesis have a maximum of 16 amino acids, so they most likely tend to occur in the form of a random coil (e.g., salmon calcitonin has 32 amino acids and in random coil conformation occurs in aqueous solutions [25]). As shown in previous works [4,26,27], aldehydes in the gas phase will force peptides to have suitable structures. After interaction with an aldehyde, peptide adopts a suitable structure, allowing molecules to bind. To simulate the affinity of the peptide to the ligand, docking of the peptide to selected molecules was performed. Preliminary simulations of docking ligands to the peptide confirmed the affinity of aldehydes. These dependencies were confirmed experimentally. Measurements on actual reference mixtures confirmed the existence of characteristic interactions, established beforehand in a theoretical model. The aim of this study was to identify characteristic interactions and affinity between receptors (selected peptides) and the ligands (volatile odorants). To obtain a better stability of the receptor layer, immobilization was performed not with the whole proteins but with peptides mimicking aldehyde binding regions in a hydrophobic cavity of the HarmOBP7 protein, identified as a pheromone-binding protein (PBP) [28], present in antennae of the *Helicoverpa armigera* moth. It was suggested that this protein might contribute of the specific pheromone blend, and also is involved in binding aldehydes [29]. Four peptides of different lengths and amino acid sequences, mimicking the HarmOBP7 binding site, were used: LEKKKKDC-NH_2_, LFDSLTDLKC-NH_2_, LFDSLTDLKKKMSEC-NH_2_, and KLLFDSLTDLKKKMSEC-NH_2_. Determination of the length and sequence of the polypeptide chain enabled us to build a biosensor with defined metrological parameters, selective with respect to a selected group of volatile compounds (long-chain aldehydes). Fundamental parameters of the biosensors were described involving nine reference substances from different classes of odorous compounds: octanal, acetaldehyde, benzaldehyde, ethanol, acetone, dimethyl sulphide, trimethyl amine, and toluene. Due to potential differences between results of the molecular modelling and the experimental model, in silico investigations were correlated with the measurements of reference mixtures of selected gases.

## 2. Materials and Methods

### 2.1. Molecular Modelling

The results of preliminary studies allowed us to select four peptides for the synthesis that mapped the aldehyde binding site in the HarmOBP7 protein, which is located in the antenna of *H. armigera.* The first step was to find in databases and literature an appropriate receptor that can bind selected group of aldehydes. The basic condition for the selection of olfactory receptors (affinity to aldehydes) was achieved by HarmOBP7. In order to get some insight into the binding site of HarmOBP7 protein, its structure was homologically modelled on the basis of the template of pheromone-binding protein from *Bombyx mori* (PDBID: 1dqe) by means of the PS2 protein structure prediction server [30]. Conclusions drawn from the analysis of this model were in agreement with the previously published results of computational analysis and a series of binding experiments [29]. It was assumed that aldehyde binding in this protein was realized by forming the Schiff base through the sidechains of Lys in position 123 (Lys123). The binding pocket was additionally stabilized by electrostatic interactions between Lys124 and Asp 121. Analogously Lys125 could interact with Glu128, allowing Lys123 to interact freely with the aldehyde ligands. According, to the affinity of this protein for hydrophobic linear compounds, the binding pocket also included Leu119, Phe116, and Leu115. 

**Figure 1:**
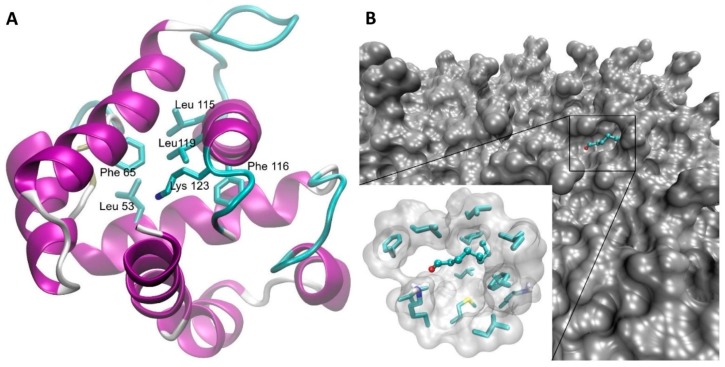
(**A**) Three-dimensional model of HarmOBP7. The model was built on the crystal structure of OBP1 of a pheromone-binding protein from *Bombyx mori* (PDBID: 1dqe). Sidechains of amino acids forming the hydrophobic binding site responsible for interaction with the long nonpolar chain of odorants as well as Lys123 participating in the Schiff base formation are shown as sticks models. (**B**) Example of octanal bound to the model of OBPP3 receptor. The ligand (octanal) is shown as a balls and sticks model, while sidechains of nonpolar amino acids (Leu8, Leu1, Leu5, Phe2, Met12) forming a hydrophobic pocket, as well as the sidechain of Lys11 putatively responsible for the Schiff base formation, are depicted as regular sticks models.

A three-dimensional model of HarmOBP7 with a binding pocket is shown in Figure 1A.

To design the peptide sequence corresponding to the binding site on the OR, short fragments near the binding pocket were chosen. The affinity energy between peptides and the aldehydes (octanal, heptanal) was determined using AutoDock to select candidates. Determination of the length of peptides was based on the calculated affinity energy. At the peptide length design stage, short fragments (5–16 amino acid residues) present in the hydrophobic cavity of the HarmOBP7 receptor were selected. A Lys residue was added to the amino acid sequence to increase the affinity of the first peptide.

At the C-terminus of each peptide chain, a Cys residue was added to allow immobilization on the gold plate of the transducer. In the original peptide sequence, position 118 was occupied by Cys. It was replaced by Ser residue [31], owing to a similar structure stabilization [32]. This type of replacement was forced with a peptide deposition on the gold surface of Quartz Crystal Microbalance (QCM). When Cys is present in the middle of the sequence of the chain and not at the C-terminus, deposition of the peptide would be difficult and unpredictable. This could negatively affect the adoption and maintenance of the appropriate peptide conformation relative to the ligand [26]. Structure determination showed that serine replaced completely the hydrogen bonds in the network. Alanine in this position was extremely destabilizing.

Models of the structures of all designed peptides were prepared on the bases of their sequences by means of the PEP-FOLD server [33,34]. As expected, the peptides adopted mostly helical structures. To create the models of sensors for affinity prediction, resulting structures of all OBPPs were multiplicated along the plane orthogonal to the axis defined by the peptide’s helix and aligned as a monolayer after random rotation of each individual peptide molecule around this axis. To simulate adsorption to the gold plate, positions of the cysteine sulfhydryl groups were restrained to the XZ plane. The obtained models of the 3 sensors were then subjected to the 100 ns molecular dynamics simulations performed with the GROMACS 4.6.7 software and the G43a2 forcefield [35]. The resulting trajectory was clustered, and the 3 most representative geometries were selected as receptors for subsequent docking calculations.

Models of all odorant molecules (ligands for docking calculations) were built by means of the HyperChem software. All dockings were then performed with the Autodock 4.2 package and all necessary parameter files, as well as ligand and receptor structures, were prepared and processed with the Autodock accompanied scripts [36]. The modified Autodock forcefield was used for binding affinity estimation, since our preliminary calculations have shown that it is capable to better recreate the experimental affinities than the original Autodock forcefields [37]. The size of the docking grid was set in such a way that it covered the central part of the peptide monolayer and extended 45 × 30 × 45 angstroms in the x, y, and z directions, respectively. For each ligand–receptor pair, 100 docking runs were performed, and the minimum energy poses were selected as the final results.

### 2.2. Peptide Synthesis and Deposition

To test the working hypotheses that emerged from the in silico analysis, OBPPs (OBPP1–LEKKKKDC-NH_2_, OBPP2–LFDSLTDLKC-NH_2_, OBPP3–LFDSLTDLKKKMSEC-NH_2_, and OBPP4–KLLFDSLTDLKKKMSEC-NH_2_) were synthesized by a standard solid-phase method using the Fmoc chemistry strategy and deposited on piezoelectric sensors (see Supplementary Materials). Purified fractions (>98% purity by HPLC) were collected, lyophilized, and stored in a refrigerator (5 °C) before deposition. Purity of the peptide solutions was checked before the immobilization process (Figure S1).

One of the advantages of the gold–alkylthiolate layer is that it is stable when exposed to air for several months [38]. The reason for this could be ascribed to the fact that immobilization on gold surfaces adsorb very strongly due to the formation of a covalent bond between the gold and the sulphur atoms. Selected 10 MHz piezoelectric electrodes (openQCM, Napoli, Italy) consisting of a quartz plate (diameter 13.7 mm) with polished Au films (diameter 5.1 mm) were prepared as previously reported [4]. The dipping process for OBPP1 was performed on both sides using a commercial dipping machine (LP100dip, Kamush, Gdańsk, Poland) with the chamber saturated with nitrogen to prevent peptide oxidation and disulphide bond formation. The changes of frequency of the QCM response signal before and after dip coating allowed assessment of the deposition level. All reagents and the volatile compounds were purchased from Sigma-Aldrich (Sigma Aldrich Co., St. Louis, MO, USA). The nine volatile compounds were of analytical grade.

### 2.3. Gas Mixtures and Measurements Setup

The reference mixtures of odorous substances were prepared in Tedlar^®^ bags using a gas mixtures generator. According to a previously reported method [4] all gas mixtures were prepared and analyzed. All concentrations presented in this paper are given in volume fractions (volume of gaseous compound in volume of dry air at 25 °C) (see Supplementary Materials, Figure S6). 

**Figure 2:**
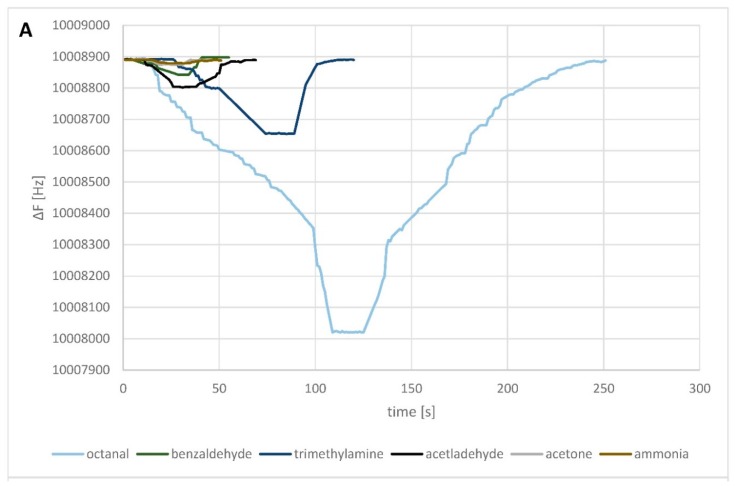

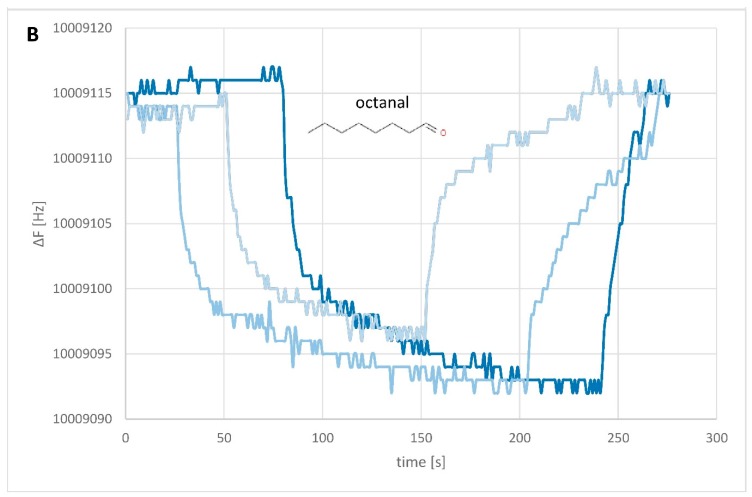
(**A**) Resonant frequency responses of the OBPP4-based biosensor to different tested compounds in high concentrations (octanal, 1435 ppm; benzaldehyde, 2198 ppm; trimethylamine, 1594 ppm; acetaldehyde, 4007 ppm; acetone, 3028 ppm; ammonia, 9619 ppm *v*/*v*) in the gas phase. (**B**) OBPP4-based biosensor responses for the lowest concentration of octanal in gas phase in three repetitions (37.5 ppm *v*/*v*).

Low-pressure pumps were used to transport gas samples to the PTFE chamber (65 cm^3^), where the biosensor was placed in a holder. Pure air (2% ± 1% relative humidity) was employed as a carrier gas. The biosensor response was stated as the frequency shift. The resonant frequency was measured with an accuracy of ±1 Hz. Signals obtained from the system were stored on the computer and processed by QCMmeter software (Gdansk University of Technology). Biosensor measurement time, in the case of the OBPP4-based biosensor for 37.5 ppm, *v*/*v* octanal, was around 300 s, composed of (i) baseline stabilization (30 s), (ii) gas introduction and adsorption (100 s), (iii) signal stabilization- signal plateau (30 s), and (iv) return to the initial sensor (150 s). Particular measurement stages differed depending on a degree of gas molecules adsorption (Figure 2). The QCM was exposed to the carrier gas after absorption of each analyte. The backshift of the crystal frequency to its initial value was taken as an indication of complete desorption.

The analytical data were characterized by the average frequency difference between the value recorded as baseline before the affinity reaction and the final value (ΔF = F_I_ – F_II_). The average was automatically calculated between 10 measurements by the software of the piezoelectric system. In all the measurements, the response of a bare QCM was monitored to confirm the negative response without peptides.

## 3. Results and Discussion

### 3.1. Peptide-Based Sensor Film Characterization

The peptide concentration used for the dipping processes was 20 mg·mL^−1^ with a volume of 7 mL. Estimated dwell time was 300 s, with an immersion rate of 240 mm·min^−1^, with the same withdraw speed. The total time of the dip coating process was 6 h. Limitation of oxygen access during deposition process, due to placing the device inside a Plexiglas chamber, allowed reduction of the peptide concentration as compared to the previous results [4], which was necessary for effective immobilization (20 mg·mL^−1^) (Figure S4). Deposition in a nitrogen atmosphere limited the formation of trimers, which contributed to a higher efficiency of binding of cysteine on the gold surface. Earlier optimization of the deposition technique provided the biosensors with high repeatability. Surface characteristics of deposited films were presented on AFM images (Figure S3) obtained by using the tapping AFM mode (AFM Ntegra Prima, NT-MDT, Moscow, Russia). The images suggest that the peptide film was visible on the transducer surface. Irregular ring structures of clusters of local aggregations of the immobilized peptides were seen for all OBPPs’ gold transducer surfaces. The distribution of peptide molecules was not uniform for all the four peptides with aggregates of different sizes and forms. A higher peptide density was obtained for OBPP3 and OBPP4 biosensors, as compared to those of OBPP1 and OBPP2 ones, which was confirmed by results from frequency changes after deposition. The deposition level of the receptor layer was calculated based on a difference in frequency before and after deposition, following the Sauerbrey equation [39], taking into account the molecular mass of each peptide (see Supplementary Materials). The lowest molar threshold for the QCM measurements was obtained for OBPP4, at approximately 1.15 nmol cm^−2^ (Figure S5).

### 3.2. Virtual Docking

Since the structure adopted by a particular peptide forming receptor’s surface is unknown, three of the most representative structures from MD trajectory were selected. After performing test docking simulation, we found that the differences of odorant molecules’ affinities to the three versions of receptors were minor and within the range of Autodock’s binding energy estimations accuracy, so only the structure representative for the most abundant cluster was chosen for all other simulations and analyses. The affinities to the model receptors resulting from docking calculations and expressed as binding energies, as reported by the Autodock software, are shown in Table 1.

For all the peptides, and particularly the two longest ones, the general bias in binding the aldehydes with long chains is visible (octanal, benzaldehyde), with ammonia, ethanol, dimethyl sulphide, and trimethyl amine on the other side of the affinity spectrum. Detailed analysis of the binding sites on the modelled receptors’ surfaces reveals that the sidechains of hydrophobic amino acids tends to form irregular pockets in the receptor’s structure, thus binding the nonpolar part of large odorant molecules, while their carbonyl group can still form the Schiff base with the ε-amine moiety of a lysine (Figure 1B). However, since the structure of this cavity is not well defined, there does not seem to be a significant difference in specificity and affinity toward particular receptors.

### 3.3. OBPPs-Based Biosensor Parameters

Computer simulations of possible interactions and affinities between ligand and peptide does not guarantee that a similar relationship would occur in practice. Ligand/peptide interaction requires a few factors in order to obtain the so-called “olfactory response” including (i) proper location of the ligand inside the pocket, (ii) suitable orientation of the ligand, (iii) stabilization of the peptide structure and bonding site, (iv) H-bond formation between suitable groups (in the case of aldehyde binding, it is formation of the Schiff base), and (v) proper distance between the binding groups. These parameters, including free binding energy, are indispensable to activate and generate signal [40]. Due to potential differences in the results of molecular modelling, the next stage of investigation involved measurements of actual solutions of selected gas molecules. Analysis of sensitivity and selectivity was then performed to verify the simulation results.

To evaluate sensitivity of the OBPPs-based biosensors, the measurements were performed for the odorous substances at different concentration levels (from ca. 50 to 9600 ppm *v*/*v*). Sensor responses to the target compound (long chain aldehyde–octanal) and to the compounds from the remaining classes (acetaldehyde, benzaldehyde, ethanol, acetone, dimethyl sulphide, trimethyl amine, and toluene) were obtained. The results indicate that the adsorption of octanal gas molecules on the OBPP4 is more favorable than that on other biosensors, representing a superior selectivity (Figure 2A). The adsorption strength of octanal molecules on the surface is useful to obtain a high response and lower detection limit. However, the main issue arising from this characteristic is the longer adsorption/desorption time, leading to higher recovery times. It could be stated that for measurements of lower concentrations of gas molecules, adsorption time (ca. 100 s) is shorter than that required for the desorption (ca. 50 s) of target gas molecules (Figure 2B). 

Humidity has only a slight influence on the piezoelectric sensor response. The measurements were conducted for all sensors and all tested VOCs for moist air with 43.5% relative humidity and compared with those taken in dry air. Observed change of frequency did not exceed ±4 Hz for the OBPP1 sensor. In case of the remaining biosensors, the changes of frequency caused by water adsorption were at the level of ca. 10 Hz. The signal recorded in the absence of the analyte (blank) signal enabled to estimate noise level. With low moisture samples (RH = 2% ± 1%), the sensor noise level was insignificant (±1 Hz). The LOD (limit of detection) was defined manually as the minimum concentration of analyte that produced a clear-cut peak of signal-to-noise ratio (S/N) [41]. Choosing the S/N ratio equal to 3:1 was considered as acceptable for estimating the LOD. Signal drift for particular sensors was taken into account upon LOD evaluation. Relative sensitivity values were employed to assess selectivity of the biosensor with respect to tested odorous molecules (Table S1). The highest selectivity coefficients were obtained for octanal (more than 3000). Relatively high values were also observed for other aldehydes (i.e. acetone, acetaldehyde and benzaldehyde). Sensors OBPP1 and OBPP2 presented high selectivity for triethylamine with reference to aldehydes. 

The metrological parameters for all the fabricated sensors were performed. For each odorous compound the following parameters were determined: sensitivity (S), limit of detection (LOD), and coefficient of determination calculated for static characteristics of the sensors. The data presented in Table 2 shows clearly that the OBPP4 sensor, immobilized with the KLLFDSLTDLKKKMSEC peptide, exhibited the highest sensitivity with respect to octanal. The remaining gases used as the interferents gave much lower responses. The OBPP4 sensor exhibited the best repeatability (RSD <= 3%), even for low concentrations of octanal (Table S2). The repeatability of OBPP1, OBPP2, and OBPP3 determined for the lowest octanal concentration (37.5 ppm *v*/*v*) was higher than 10% due to its high detection limit (higher than presented concentration).

According to calibration curves for octanal (static mode) presented in Supplementary Materials (Figure S7), determination coefficients for OBPP2, OBPP3, and OBPP4 were higher than 0.97, which indicates a very good linear dependence of the signal on the analyte concentration. In the case of the OBPP1 sensor, its determination coefficient was around 0.94. This value, as well as the position of the measuring points, suggests a nonlinear course of the static characteristics of the sensor.

## 4. Conclusions

**Figure 3:**
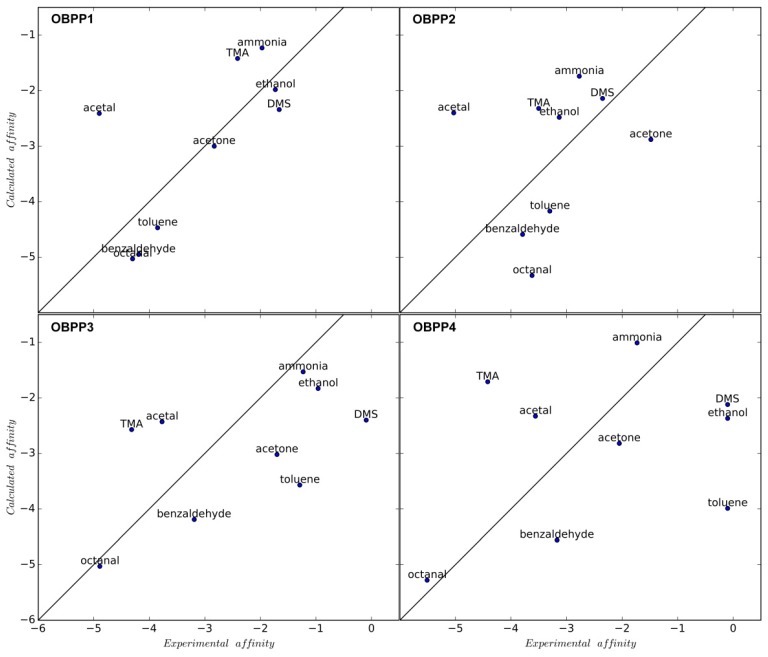
Correlation between experimental and calculated affinities for OBPP1, OBPP2, OBPP3, and OBPP4. The solid lines represent a perfect agreement between the two values. The calculated Pearsons’ correlation coefficients are 0.69, 0.22, 0.56, and 0.33 respectively. The calculated affinity is given as reported by Autodock’s binding affinity, and the experimental affinity value is calculated as 0.8*ln(∆F).

Our results show that for longer peptides, in-silico prediction of preferential binding of octanal molecule was achieved, while for shorter ones predicted binding of acetal was heavily underestimated (Figure 3). Most probably it is connected with the already-mentioned difficulty of shorter peptides to take well defined structure and the favored acetal binding via the Schiff base formation, without additional specific interactions with the receptor. That phenomenon escapes the interaction model implemented in the Autodock, and is responsible for this underestimation of acetal binding energy. For longer peptides, relatively stable hydrophobic pockets just start to be formed, which are capable of binding of nonpolar ligand parts. This is reflected in an increased affinity of these ligands both in calculation results as well as sensor responses (Figure S7). Experimental verification of in silico investigation results confirm that the most effective interaction with the ligand is exhibited by the longest peptide (OBPP4). Longer adsorption time for the OBPP4-based biosensor suggests other types of adsorption and interaction of the compounds with the receptor layer via different mechanism [42]. Only some of these are linked to specific functionalities of the receptor layer (e.g., interactions of the volatiles with carbonyl or hydroxyl groups of the sidechains of amino acids). In the piezoelectric sensors, there is no possibility to differentiate between particular types of specific and nonspecific interactions. Hence, the QCM biosensor response can be regarded as a combination of nonselective and selective interactions, conditioned by functional groups of ligands. Only the increased sensors’ sensitivity and longer adsorption time can suggest a Schiff base formation as in the case of the calculation model. A similar phenomenon was observed for metallo-porphyrins [43] and peptides [44]. The results of those investigations can facilitate application of synthetic peptides, mimicking ligand binding sites in OBPs, as the receptor elements of odor biosensors. The effect of the research is the selection of the peptide selective with respect to the octanal molecule (a volatile lung cancer biomarker and indicator of edible oils’ stability), which can be useful in potential applications. Additionally, a dependence between chain length and receptor/ligand affinity was determined. Similar sensors for aldehydes detection was developed by Ko et al. [45], where olfactory an receptor protein of rats, I7, was expressed on the plasma membrane of human embryonic kidney (HEK)-293 cells. The aldehydes were detected at low as 10^−8^ mM. They [46] presented that using planar microelectrodes, electrical signals could be obtained from HEK293 cells expressing ORI7 and the olfactory signals could be enhanced by electrical stimulation. They demonstrated real-time monitoring of cells’ responses in the liquid phase to odorants (heptanal, octanal, nonanal, decanal, and helional) stimuli using SPR (Surface Plasmon Resonance). However, difficulties with deposition of the cells and thus short lifespan of the sensors remain unsolved.

A peptide receptor must possess appropriate spatial structure to bind ligands effectively. Receptor/ligand interactions in the gas phase can force the attaining of a proper structure which enables effective binding. The peptide with the longest amino acid sequence (OBPP4) adopts an ordered form in solution, whereas after immobilization on a transducer this order is disturbed. Irregular cluster structures are preferred, nevertheless, selective detection of odorant molecules is possible. The ease of peptides synthesis via modification of sidechains of amino acids allows simple improvement of affinity with respect to particular odorants. Intervention into peptide structure, via implementation of suitable linkers, spacers, and stabilizing structures, can have positive influence on the attaining of a desired spatial structure. A bigger distance between the binding pocket and secondary transducer substrate combined with structure enforcement, for example with sulphur bridges, can have beneficial effect on receptor/ligand affinity. Elimination of, or a decrease in, signal drift connected with moisture content in samples can enable application of the biosensors for this type of samples. Still there is a need for research on selectivity and specificity of peptides with respect to long-chain aldehydes, as well as improvement of biosensor parameters through structure stabilization during receptor/ligand binding. Nevertheless, the designed peptide, derived directly from the OBP binding site, confirmed their high applicability in gas biosensors. In addition, the peptide-based biosensor showed a high selectivity to octanal. The peptide-based biosensors offer a promising approach for chemical molecular gas phase sensing, as well as for binding functions investigation of such biosensors.

## Figures and Tables

**Table 1:** The affinity levels of peptide-based biosensors vs. volatile compounds.

Compound	OBPP1 [kcal/mol]	OBPP2 [kcal/mol]	OBPP3 [kcal/mol]	OBPP4 [kcal/mol]
Octanal	−5.03	−5.33	−5.03	−5.28
Acetaldehyde	−2.41	−2.40	−2.43	−2.33
Benzaldehyde	−4.95	−4.59	−4.19	−4.56
Ethanol	−2.55	−2.48	−2.48	−2.37
Acetone	−3.00	−2.88	−3.02	−2.82
Dimethyl Sulphide	−2.34	−2.14	−2.40	−2.12
Trimethyl Amine	−1.42	−2.32	−2.57	−1.71
Toluene	−4.47	−4.17	−3.57	−3.99
Ammonia	−1.23	−1.74	−1.53	−1.01

**Table 2:** Comparison of biosensors’ responses to different odorants.

Compounds	Parameters	OBPP1	OBPP2	OBPP3	OBPP4
Ammonia	S·10^3^ [Hz/ppm]	0.7	4.9	0.1	0.5
	LOD [ppm v/v]	>1500	>1500	>1500	>1500
	R2	0.92	0.95	0.313	0.661
Acetone	S·10^3^ [Hz/ppm]	16.3	2.6	3.3	4.0
	LOD	>1500	>1500	>1500	>1500
	R2	0.939	0.818	0.801	0.771
Dimethyl sulphide	S·10^3^ [Hz/ppm]	2.0	9.7	no response	no response
	LOD	>1500	>1500	no response	no response
	R2	0.458	0.951	no response	no response
Acetaldehyde	S·10^3^ [Hz/ppm]	116.8	142.8	29.1	27.5
	LOD	243.4	574.0	571.1	>1500
	R2	0.998	0.987	0.987	0.987
Ethanol	S·10^3^ [Hz/ppm]	2.3	13.5	no response	no response
	LOD	>1500	1224.0	no response	no response
	R2	0.751	0.939	no response	no response
Trimethylamine	S·10^3^ [Hz/ppm]	20.9	53.7	145.6	159.6
	LOD	>1500	424.5	175.5	105.1
	R2	0.96	0.957	0.992	0.997
Benzaldehyde	S·10^3^ [Hz/ppm]	91.5	55.8	26.1	25.0
	LOD	678.6	650.3	892.0	485.3
	R2	0.943	0.947	0.905	0.970
Toluene	S·10^3^ [Hz/ppm]	63.3	31.3	no response	no response
	LOD	906.2	826.3	no response	no response
	R2	0.895	0.911	no response	no response
Octanal	S·10^3^ [Hz/ppm]	16.4	67.7	335.4	667.5
	LOD	455.6	293.9	48.5	37.5
	R2	0.940	0.974	0.999	0.997

## Supplementary Materials

Supplementary materials can be found at https://www.mdpi.com/1424-8220/19/19/4284/s1.
