# Supplementary material for: A Highly Selective Biosensor Based on Peptide Directly Derived from the HarmOBP7 Aldehyde Binding Site
Source: Sensors (Basel). 2019 Oct 3;19(19):4284. doi: 10.3390/s19194284 (PMC6806164; doi:10.3390/s19194284)
Supplement: Supplementary file 1 [file sensors-19-04284-s001.pdf]

Supplementary Materials

A Highly Selective Biosensor Based on Peptide Directly Derived from the HarmOBP7 Aldehyde Binding Site

Tomasz Wasilewski [1,*], Bartosz Szulczyński [2], Marek Wojciechowski [3], Wojciech Kamysz [1] and Jacek Gębicki [2,*]

[1] Department of Inorganic Chemistry, Faculty of Pharmacy, Medical University of Gdańsk, Hallera 107, 80-416 Gdansk, Poland; kamysz@gumed.edu.pl
[2] Department of Process Engineering and Chemical Technology, Chemical Faculty, Gdańsk University of Technology, Narutowicza 11/12, 80-233 Gdańsk, Poland; bartosz.szulczynski@pg.edu.pl
[3] Department of Pharmaceutical Technology and Biochemistry, Chemical Faculty, Gdansk University of Technology, Narutowicza 11/12, 80-233 Gdańsk, Poland; marek.wojciechowski@pg.edu.pl
* Correspondence: tomasz.wasilewski@gumed.edu.pl (T.W.); jacek.gebicki@pg.edu.pl (J.G.)

Received: 8 September 2019; Accepted: 1 October 2019; Published: 3 October 2019

Peptides Synthesis and Deposition

Synthesis of the amides was carried out automatically on a microwave Liberty Blue™ Automated Microwave Peptide Synthesizer (CEM Corporation, Mathews, NC, USA), with an IR temperature sensor and a gas cooling system. The elongation of the peptide chain was carried out in consecutive cycles of deprotection and coupling. After the synthesis, the peptide resin was dried under vacuum. Cleavage from the resin was accomplished in TFA using a scavengers mixture – TFA:EDT:TIS:water, 94:2:2:2 (v/v/v/v) for 90 min with stirring. The peptide was precipitated with cold diethyl ether and lyophilized. Subsequently, the peptides were purified by reversed-phase high-performance liquid chromatography (RP-HPLC) with LP-chrom software. The crude and pure peptides were analysed by HPLC in a water/acetonitrile gradient and purified on a Waters X-Bridge Prep C18 column. Purity was confirmed by HPLC (Varian, Mulgrave, VIC, Australia) (Figure S1.a)

Pure ractions (> 95%, by HPLC analysis) were collected and lyophilized. The molecular weights were confirmed by ESI-MS (Waters Acquity SQD, Milford, MA)(Figure S1.b).

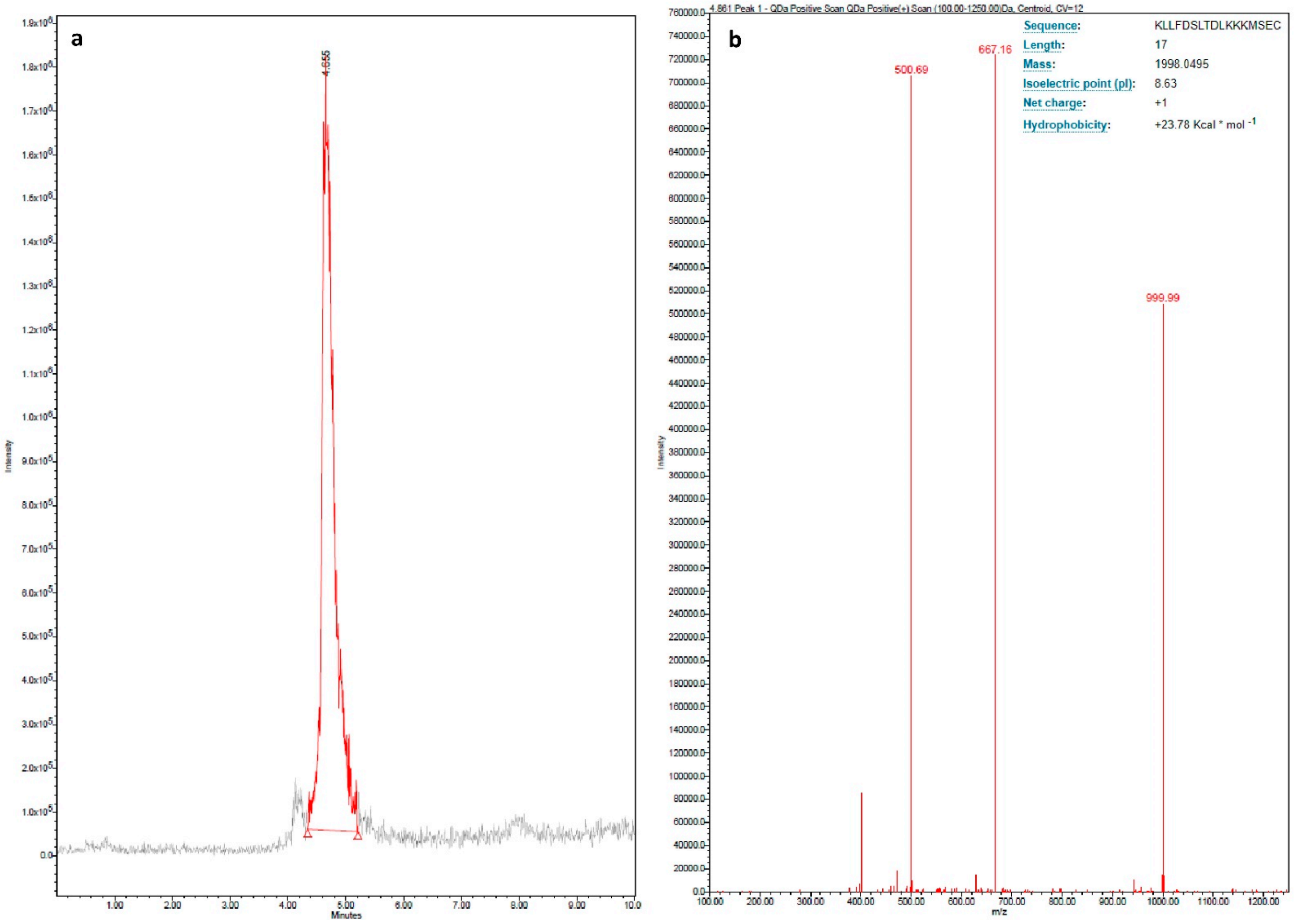

Figure 1. (a) HPLC-UV/VIS chromatogram and (b) mass spectrum of purified KLLFDSLTDLKKKMSEC-NH_2.

Schematic presentation of synthetic peptides based sensor fabrication was presented in Figure S2.

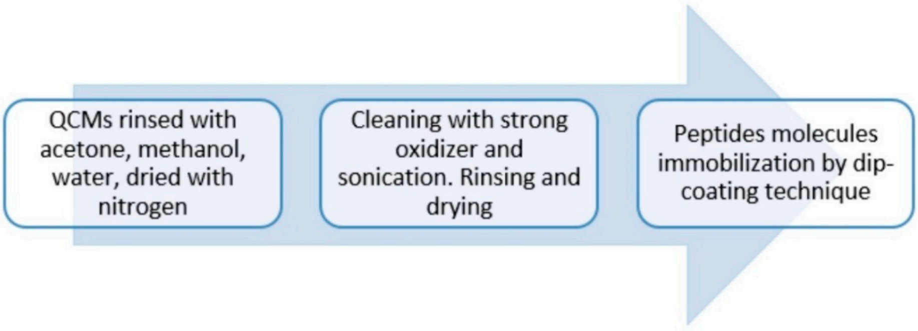

Figure 2. Scheme of steps concerning piezoelectric transductor preparation.

Because of Cys- terminated sequence, the peptides possess an ability to form self-assembling layers on the gold electrode surface. Deposition process occurs spontaneously due to chemisorption

of the modifying compound on metal. The sensor surface was automatically dried and rinsed between the immersion cycles. Modification of the sensing surface is an important process in QCM biosensor studies. Peptide deposition generates a suitable recognition layer with a specific property or function. The surface of QCM crystals was rinsed with acetone, methanol and deionized water, then dried with nitrogen until a final solvent evaporation from the surface. A strong oxidizer (Pyranha solution, 30 % H_2O_2:H_2SO_4, 1:3, v/v) was placed on the gold electrode to remove organic materials. Next step of the QCM preparation was rinsing with deionized water and ethanol and drying with nitrogen. Every deposition process was carried out in ambient temperature in the dark to avoid possible peptide degradation.

Deposition of a mass on a thin gold surface induces a decrease in the resonant frequency for a rigid substance. A relationship between frequency change and peptide deposition efficiency is expressed by the Sauerbrey [1] (1):

$$\Delta f = \frac{-2\Delta m f_0^2}{\left[A\left(\rho_q \mu_q\right)^{1/2}\right]} \tag{1}$$

where, ρ_q and μ_q are the density (2.648 g·cm^{-3}) and shear modulus of quartz (2.947×10^{11} g·cm^{-1}·s^{2}), respectively f_0 is the crystal fundamental frequency of the piezoelectric quartz crystal, A is the crystal piezoelectrically active geometrical area which is defined by the area of the deposited metallic film on the crystal, Δm and Δf are the mass and system frequency changes. According to this equation, the mass of a thin layer deposited on the surface can be calculated through measuring the changes in the resonant frequency. For a typical piezoelectric sensor with a 10 MHz frequency, a change in mass of 4.4 ng results in a frequency change of around 1 Hz·cm^{-2}.

The AFM images were acquired to examine the morphology of the film surface on the peptide-based biosensors. An AFM Ntegra Prima device manufactured by NT-MDT (Moscow, Russia) was used. In the topographic measurements, the tapping mode with the set-point equal to half-value of free oscillations amplitude was applied. The measurements were carried out using conductive probes of the NSG 01 type, manufactured by NT-MDT. The geometric dimensions of the probe lever were 125 × 30 × 2 (L × W × T/mm), while other parameters were as follows: resonance frequency: 150 kHz, spring constant: 5.1 N/m, radius of tip curvature: 10 nm. In all studies on biosensors, regardless of the deposition method, a peptide monolayer was observed (10 μm × 10 μm). To show the uniformity of the layers an AFM images at 10 μm × 10 μm with basic parameters and 1 μm × 1 μm are presented in Figure S3.

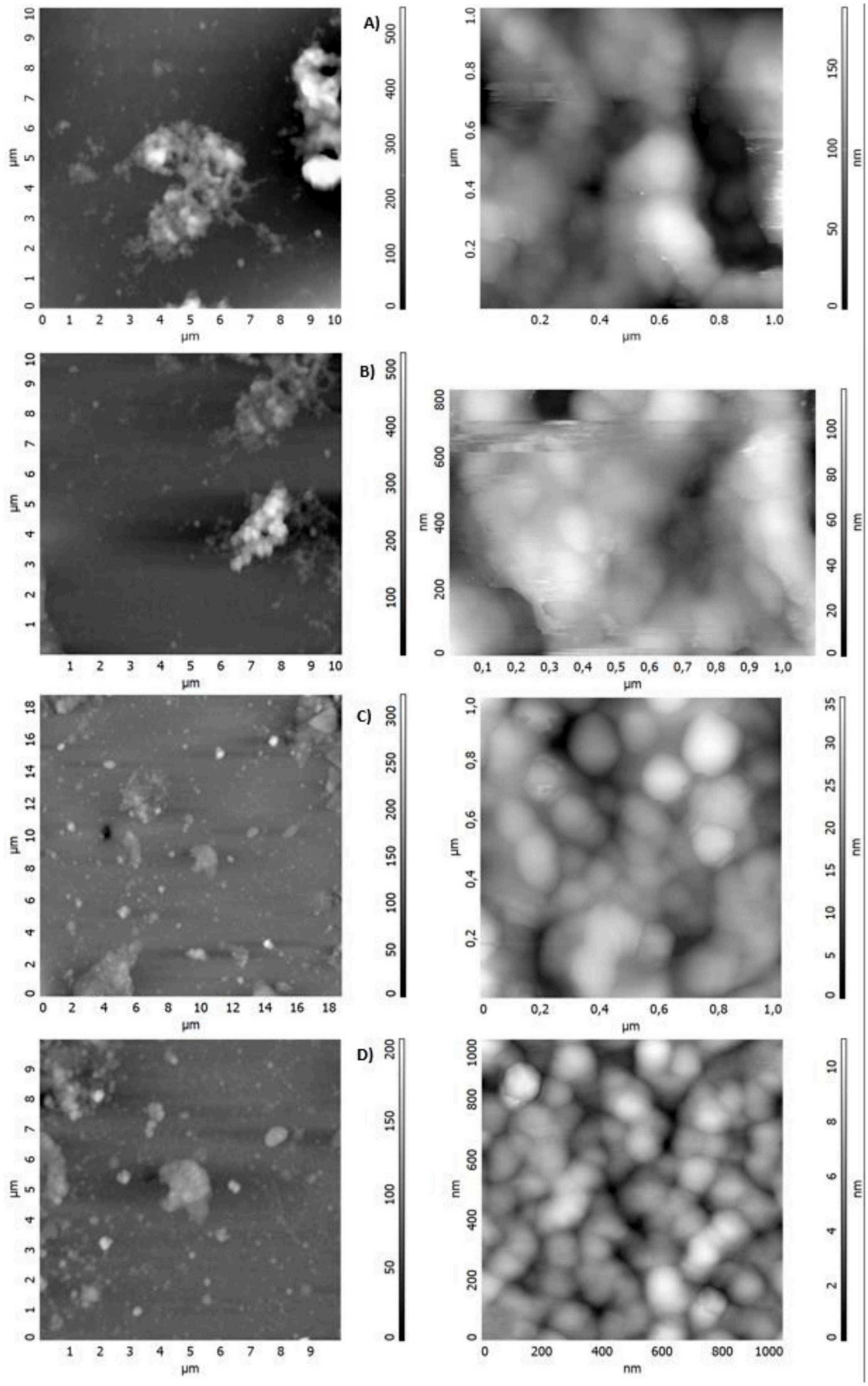

Figure 3. AFM images of fabricated sensors: (A) OBPP1 (Peak-to-peak, Sy: 1128.98 nm; Ten point height, Sz: 557.869 nm, Average Roughness, Sa: 161.4 nm); (B) OBPP2 (Sy: 528.02 nm, Sz: 263.18 nm, Sa: 24.30 nm); (C) OBPP3 ((Sy: 211.73 nm, Sz: 105.88 nm, Sa: 33.73 nm); (D) ((Sy: 206.19 nm, Sz: 98.65 nm, Sa: 8.0 nm).

Average depositions were calculated for three sensors of each dip-coating. Saturation occurred at 20 mg·mL^{-1}, this concentration was chosen for peptide deposition using dip-coating technique. In Figure S4, shifts in frequency during deposition cycles in different concentrations are presented.

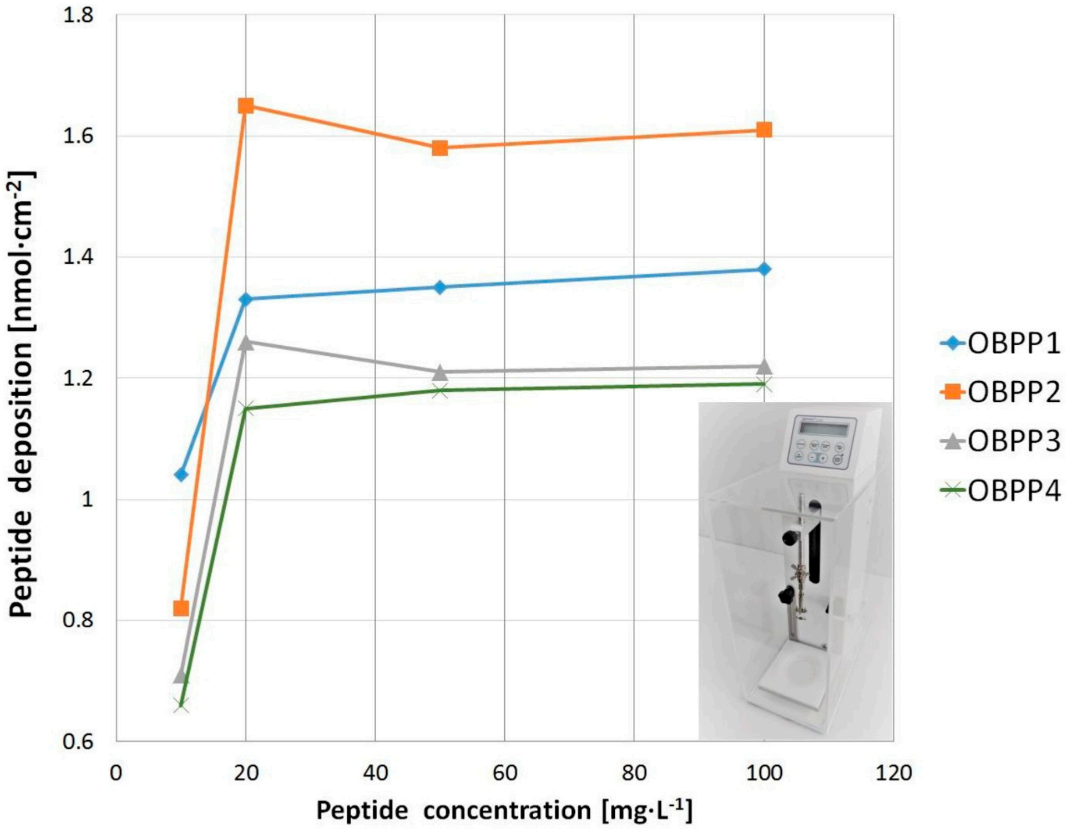

Figure 4. Comparison of frequency shifts during deposition by using dip-coater with chamber filled with an inert gas.

The correlation between calculated and measured concentrations of gas mixtures are presented on Figure S4.

According to previously reported evaluation of most effective peptide deposition method, dip-coating was selected to obtain homogenous biosensors receptor layers. The first peptide (LEKKKKDC) was prepared in water/acetonitrile (1:1, *v*/*v*,), whereas the remaining three LFDSLTDLKC, LFDSLTDLKKKMSEC, KLLFDSLTDLKKKMSEC in acetic acid/acetonitrile (9:1, *v*/*v*) allowing complete dissolution of the compounds. Additionally, the compounds were degassed with helium (10 min) before use in order to prevent oxidation of thiol group (−SH) and formation of sulphur bridges (–S–S). Dip-coating processes were conducted at room temperature, in darkness.

Average deposition on electrode surface for four peptide-based sensors, acquired in three repetitions, is shown in Figure S5.

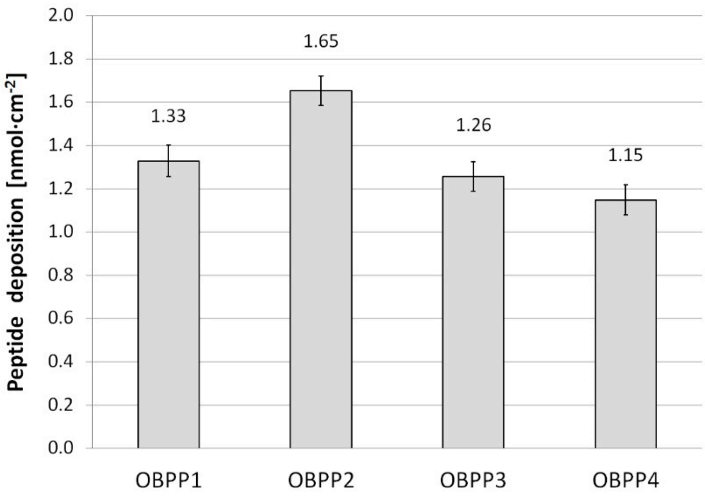

Figure 5. The average deposition of four biosensors, determined for three repetitions.

Gas Mixtures and Measurements Setup

Correctness of the prepared reference solutions was verified using gas chromatography coupled with thermal conductivity detector (AutoSystem XL, PerkinElmer) and flame ionization detector (430-GC, Bruker®, Bremen, Germany), which is confirmed by presented correlation plots (Figure S5) and high coefficients of determination $R^2 > 0.97$ (Figure S5).

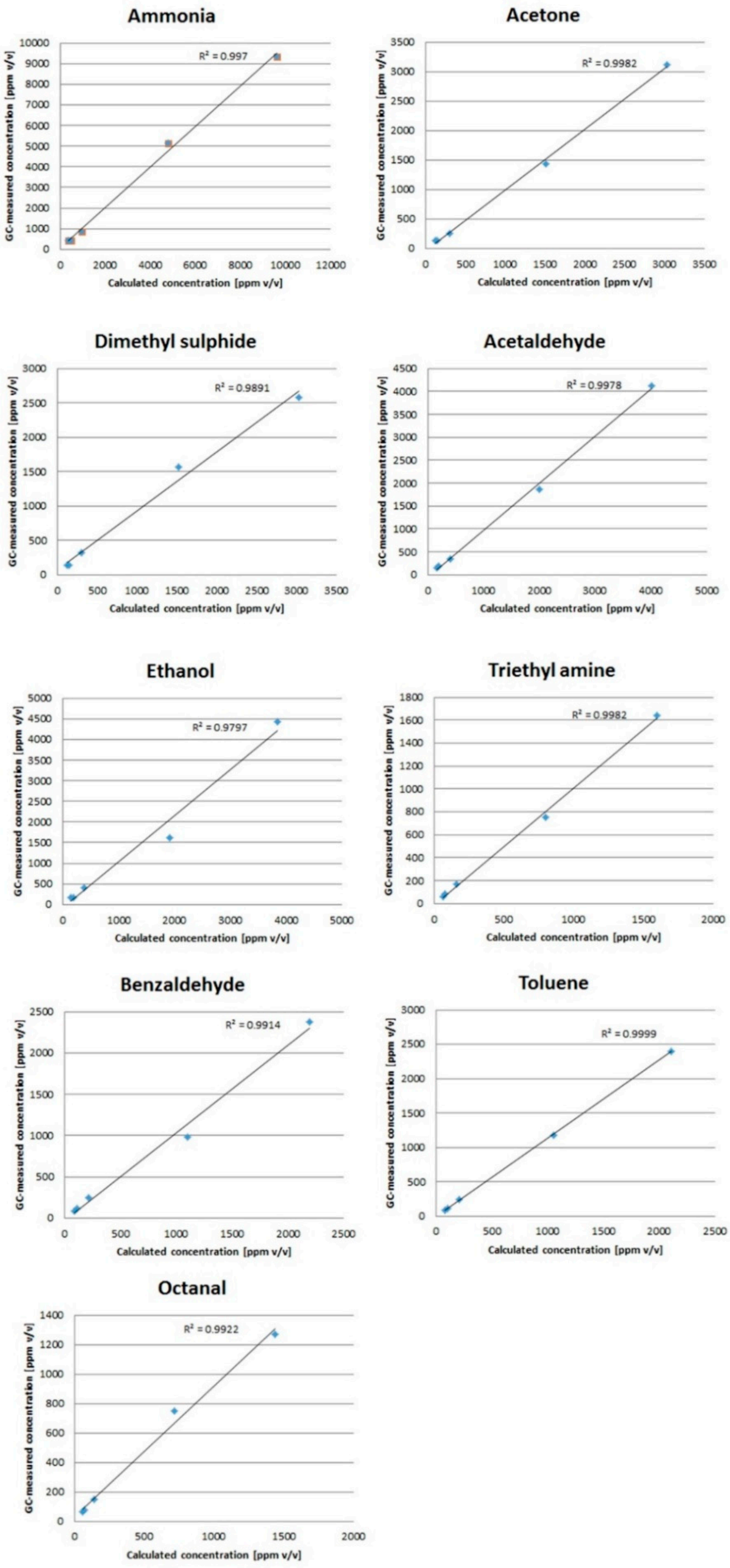

Figure 5. Correlation plots showing the correctness of gas mixtures preparation.

OBPPs- Based Biosensors Parameters

Table S1 presents the values of selectivity coefficients of all sensors. The selectivity coefficients were calculated using Formula (1):

$$k = \frac{S_{Compound\ A}}{S_{Compound\ B}} \tag{2}$$

where: k – selectivity coefficient [-], $S_{compound\ A,B}$ – sensor sensitivity for selected odorous compound A and B (Table S1).

Table 1. Selectivity coefficients (k) of all fabricated sensors.

**OBPP1**		Compound B								
		ammonia	acetone	dimethyl sulfide	acetaldehyde	ethanol	triethylamine	benzaldehyde	toluene	octanal
Compound A	ammonia	1.00	0.043	0.350	0.006	0.304	0.033	0.008	0.011	0.004
	acetone	23.286	1.000	8.150	0.140	7.087	0.780	0.178	0.258	0.099
	dimethyl sulfide	2.857	0.123	1.000	0.017	0.870	0.096	0.022	0.032	0.012
	acetaldehyde	166.857	7.166	58.400	1.000	50.783	5.589	1.277	1.845	0.712
	ethanol	3.286	0.141	1.150	0.020	1.000	0.110	0.025	0.036	0.014
	triethylamine	29.857	1.282	10.450	0.179	9.087	1.000	0.228	0.330	0.127
	benzaldehyde	130.714	5.613	45.750	0.783	39.783	4.378	1.000	1.445	0.558
	toluene	90.429	3.883	31.650	0.542	27.522	3.029	0.692	1.000	0.386
	octanal	234.286	10.061	82.000	1.404	71.304	7.847	1.792	2.591	1.000
**OBPP2**		Compound B								
		ammonia	acetone	dimethyl sulfide	acetaldehyde	ethanol	triethylamine	benzaldehyde	toluene	octanal
Compound A	ammonia	1.000	1.885	0.505	0.034	0.363	0.091	0.088	0.157	0.072
	acetone	0.531	1.000	0.268	0.018	0.193	0.048	0.047	0.083	0.038
	dimethyl sulfide	1.980	3.731	1.000	0.068	0.719	0.181	0.174	0.310	0.143
	acetaldehyde	29.143	54.923	14.722	1.000	10.578	2.659	2.559	4.562	2.109
	ethanol	2.755	5.192	1.392	0.095	1.000	0.251	0.242	0.431	0.199
	triethylamine	10.959	20.654	5.536	0.376	3.978	1.000	0.962	1.716	0.793
	benzaldehyde	11.388	21.462	5.753	0.391	4.133	1.039	1.000	1.783	0.824
	toluene	6.388	12.038	3.227	0.219	2.319	0.583	0.561	1.000	0.462

	octanal	13.816	26.038	6.979	0.474	5.015	1.261	1.213	2.163	1.000

**OBPP3**		Compound B					
		ammonia	acetone	acetaldehyde	triethylamine	benzaldehyde	octanal
Compound A	ammonia	1.000	0.030	0.003	0.001	0.004	0.001
	acetone	33.000	1.000	0.113	0.023	0.126	0.010
	acetaldehyde	291.000	8.818	1.000	0.200	1.115	0.087
	triethylamine	1456.000	44.121	5.003	1.000	5.579	0.434
	benzaldehyde	261.000	7.909	0.897	0.179	1.000	0.078
	octanal	3354.000	101.636	11.526	2.304	12.851	1.000
**OBPP4**		Compound B					
		ammonia	acetone	acetaldehyde	triethylamine	benzaldehyde	octanal
Compound A	ammonia	1.000	0.125	0.018	0.003	0.020	0.001
	acetone	8.000	1.000	0.145	0.025	0.160	0.006
	acetaldehyde	55.000	6.875	1.000	0.172	1.100	0.041
	triethylamine	319.200	39.900	5.804	1.000	6.384	0.239
	benzaldehyde	50.000	6.250	0.909	0.157	1.000	0.037
	octanal	1335.000	166.875	24.273	4.182	26.700	1.000

Table 2. Repeatability of peptide-based biosensors for three selected concentrations of octanal.

	OBPP1	OBPP2	OBPP3	OBPP4
Octanal concentration	144	144	144	144
Freq mean	8.0	6.3	16. 7	123.7
Std. Dev.	2.5	2.4	2.0	3.7
RSD	30.6%	37.2%	12.3%	3.0%
Octanal concentration	717	717	717	717
Freq mean	54.3	34.7	209.3	495.0
Std. Dev.	3. 7	3.1	6.6	6.5
RSD	6.8%	8.9%	3.2%	1.3%
Octanal concentration	1435	1435	1435	1435
Freq mean	216.3	93.0	449.7	984. 7
Std. Dev.	2.6	4.3	4.5	4.6
RSD	1.2%	4.6%	1.0%	0.5%

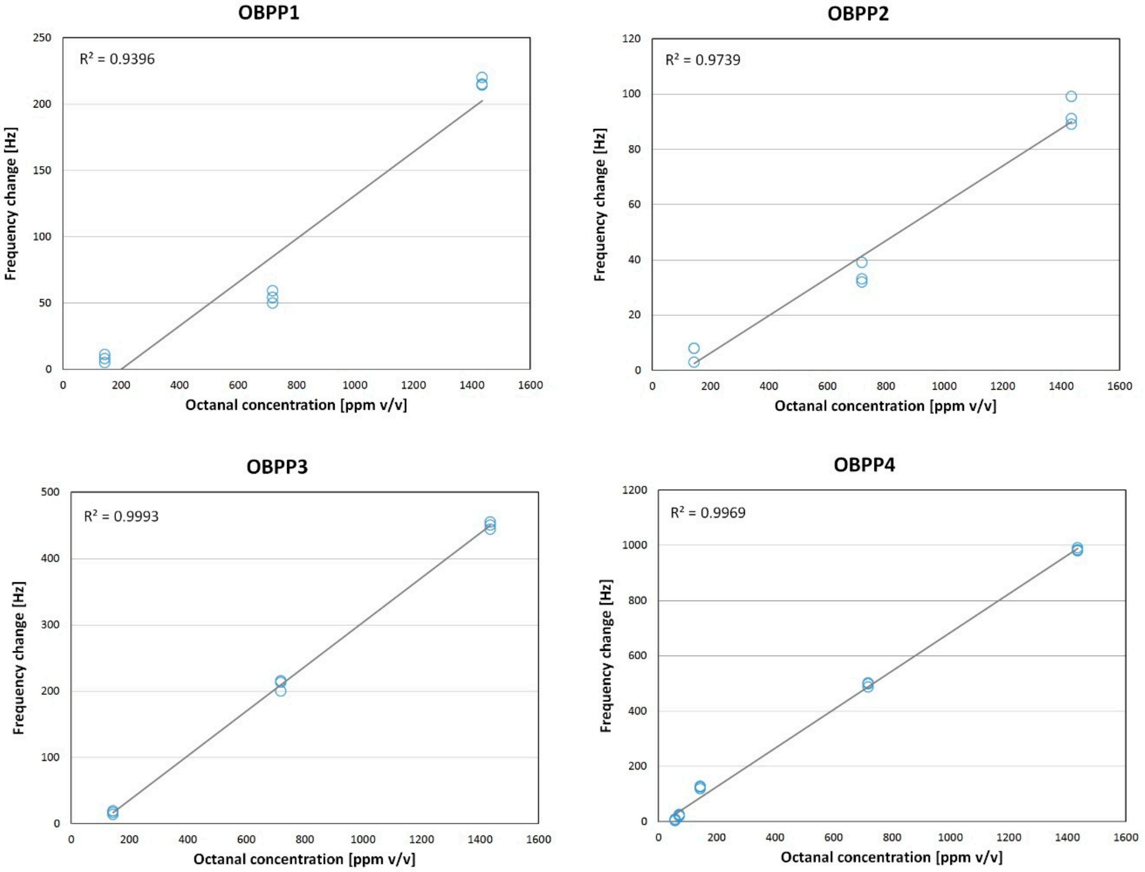

Figure 6. Calibration curves (static mode) of four peptide-based biosensors for octanal.

References

1. Latif, U.; Can, S.; Hayden, O.; Grillberger, P.; Dickert, F.L. Sauerbrey and anti-Sauerbrey behavioral studies in QCM sensors-Detection of bioanalytes. *Sensors Actuators, B Chem.* **2013**, 176, 825–830, doi:10.1016/j.snb.2012.09.064
